# Lipid metabolism of clear cell renal cell carcinoma predicts survival and affects intratumoral CD8 T cells

**DOI:** 10.1016/j.tranon.2025.102513

**Published:** 2025-09-02

**Authors:** Jakob Simeth, Simon Engelmann, Roman Mayr, Sebastian Kaelble, Florian Weber, Renate Pichler, Katja Dettmer, Peter J Oefner, Marcus Höring, Luisa Symeou, Katharina Freitag, Kilian Wagner, Maximilian Burger, Wolfgang Herr, Marina Kreutz, Rainer Spang, Gerhard Liebisch, Peter J Siska

**Affiliations:** a Chair of Statistical Bioinformatics, Institute of Functional Genomics, University of Regensburg, Regensburg, Germany; b Leibniz Institute for Immunotherapy, Regensburg, Germany; c Department of Urology, Caritas St. Josef Medical Centre, University of Regensburg, Regensburg, Germany; d Institute of Pathology, University of Regensburg, Regensburg, Germany; e Department of Urology, Comprehensive Cancer Center Innsbruck, Medical University of Innsbruck, Innsbruck, Austria; f Chair and Institute of Functional Genomics, University of Regensburg, Regensburg, Germany; g Institute of Clinical Chemistry and Laboratory Medicine, University Hospital Regensburg, Regensburg, Germany; h Department of Otorhinolaryngology, Regensburg University Hospital, Regensburg, Germany; i Department of Internal Medicine III, Hematology and Medical Oncology, University Hospital Regensburg, Regensburg, Germany

**Keywords:** Lipids, Metabolism, Renal cell carcinoma, Oncoimmunology, T cell, Prognosis

## Abstract

**Background:**

Clear cell renal cell carcinoma (ccRCC), the most common RCC subtype, both accumulates and depletes selected lipid species. However, the prognostic role of lipid metabolic reprogramming in ccRCC has not been studied in detail so far. In addition, ccRCC can show a dense immune infiltration. Intriguingly, tumor infiltration with T cells can be negatively prognostic in RCC. This comprehensive study of the transcriptome, lipidome and immune infiltrate of ccRCC tumors elucidates the prognostic role of lipid-metabolic pathways and their possible interaction with tumor infiltrating T cells.

**Methods:**

Freshly resected RCC tumors and adjacent kidney tissues and extracellular fluids were processed and subjected to mass-spectrometry based lipidomics and lipid staining (*n* = 36). Hierarchical clustering was performed using the transcriptome data and gene group definitions obtained from publicly available databases (TCGA, 526 ccRCC and 287 papillary RCC). Phenotype, activation, proliferation and fatty acid uptake were assessed in ccRCC infiltrating T cells at single cell level ex vivo (*n* = 22) or after treatment with oleate and palmitate in vitro (*n* = 4).

**Results:**

ccRCC tumors accumulated lipids, notably those containing oleate. Clustering of RCC patients based on the expression of genes involved in fatty acid degradation (FAD) and cholesterol synthesis (chol) was able to predict survival and was superior to clustering based on genes involved in fatty acid synthesis or fatty acid elongation. Further, prognostic clustering was observed in ccRCC, but not in papillary RCC tumors, and it was independent of major clinical parameters. The FAD/chol cluster with poor prognosis showed a trend toward decreased prevalence of *VHL* mutations and higher *c-MET* copy numbers. Moreover, this cluster associated with dysregulated inflammation hallmarked by low *PRF1*, but high *IFNɣ* expression. Tumor infiltrating T cells showed increased fatty acid uptake, and CD8 T cell infiltration negatively correlated with oleate-associated lipid species found in the extracellular space of ccRCC tumors. Lastly, oleate treatment ex vivo suppressed the activation and perforin production of CD8 T cells from ccRCC tumors.

**Conclusions:**

Our study describes a robust, prognostic clustering of lipid gene expression that is both ccRCC-specific and independent of major parameters such as tumor size or aggressiveness. Furthermore, we propose that oleate accumulation in the RCC lipidome affects intratumoral CD8 T cell infiltration and function.

## Highlights

• Hierarchical clustering of lipid metabolic genes allows robust outcome prediction of clear cell renal cell carcinoma (ccRCC) patients.
• ccRCC tumors accumulate storage lipids enriched in oleate.
• Fatty acid degradation and cholesterol synthesis in ccRCC tumors are associated with changes in the tumor immune infiltrate.
• CD8 T cells in ccRCC take up lipids and their frequencies correlate with the lipidome of tumor extracellular fluid.
• Oleate induces dysfunction of CD8 T cells from ccRCC tumors.

## Background

Renal cell carcinoma (RCC) tumors are highly metabolically active. The most common RCC subtype, clear cell RCC (ccRCC), is characterized by cells accumulating lipids and glycogen, morphologically appearing as “clear cells” [[1], [2], [3]]. However, the biologic and prognostic relevance of this metabolic feature remains insufficiently elucidated. While the overall concentration of free fatty acids (FA) in ccRCC tissues can be decreased [4], the FA composition of ccRCC tissues can be skewed [5], and FA-length can change with disease progression [6]. Moreover, ccRCC triglyceride (TG)-FA show a specific saturation profile [1]. On the other hand, ccRCC tumors can show high levels of cholesterol and cholesterol esters [4,7]. This ccRCC metabolic re-programming can accompany tumor evolution and spreading and has not been observed in other RCC subtypes [2,3,8].

Previous studies in ccRCC suggested a prognostic relevance of selected lipid metabolic genes such as fatty acid synthase [9,10], enoyl-CoA hydratase short-chain 1 (*ECHS1*) [11], HDL-Receptor *SR-BI* [12], the lipid transporter CD36 [13], FA catabolic enzymes *CPT1A, HADHA, HADHB* and *ACAT1* [10,14] or the fatty acid transport protein 4 [15]. Only few studies took advantage of gene sets representing defined metabolic pathways. Hakimi et al. and Liu et al. used clustering of ccRCC patients based on expression of metabolic genes with prognostic significance [6,16]. Shen et al. found a correlation of fatty acid metabolism related long noncoding RNA with survival of RCC patients [17] and Zhang et al. identified eight metabolic genes in RCC with prognostic and immunological relevance [18]. In selected tumor entities, increased lipid metabolic activity can be prognostically favorable [19]. In melanoma, increased lipid metabolism has been associated with improved response to immunotherapy due to up-regulated antigen presentation [20]. Nevertheless, the prognostic relevance of specific lipid metabolic programs of ccRCC remains insufficiently defined and it is unclear, whether changes in these programs are clear-cell RCC specific and whether they can affect cells in the tumor-microenvironment.

For several tumor entities, a positive role of T cell infiltration has been reported. Specifically, CD8 T cell tumoral infiltration is often associated with favorable outcome [21,22]. Nevertheless, CD8 T cell infiltration can also be unfavorable, such as in gastric cancer [23] and hepatocellular carcinoma, where CD8 T cells can promote tumors in metabolically dysregulated environments [24]. In addition, CD8 T cells induce immunosuppressive factors and a recruitment of regulatory T cells into melanoma tumors [25]. As opposed to other genitourinary cancers such as testicular tumors, where we previously described a positive role for CD8 T cells [26], several studies suggested a negative prognostic role of CD8 TIL in ccRCC [[27], [28], [29], [30]]. Perforin 1, a key effector molecule produced by CD8 T cells and NK cells is positively prognostic in RCC [31]. However, it is unclear whether RCC tumors modulate perforin production to impair anti-tumor immunity.

T cells are highly metabolically active and might therefore react to the availability of nutrients or metabolic products in tumors [32]. T cell stimulation leads to an increased glycolysis [33,34], but also to a down-regulation of CPT1A, which mediates long-chain-FA (LCFA) oxidation [35,36]. On the other hand, the uptake and metabolism of FA is required for T cell activation and function [37]. Nevertheless, it remains unclear, whether tumor-induced changes of the lipid-metabolic microenvironment affect T cell mediated inflammation.

Here, we exploited lipid-metabolic and inflammatory profiles of RCC tumors to discern a cluster of lipid genes that predicts outcome and suggests T cell regulation by tumor fatty acid metabolism. We also show that accumulation of oleate in RCC negatively affects CD8 T cells in vitro.

## Methods

### Tumor patients and healthy donors

Tissues from 47 patients (Suppl. Table 1) treated at the University Hospital Regensburg for renal mass were included for metabolic and in vitro studies. Collection of primary tumor samples and surrounding healthy tissue was accomplished after approval by the ethics committee (University Regensburg, reference numbers 16–355–101 and 08/108) in accordance with the Helsinki Declaration and after receipt of signed informed consent from the patients. For analyses of the tumor lipidome, kidney tissues were surgically removed and cryopreserved. To isolate tissue fluid, samples from freshly dissected tumors were processed as previously described [38,39]. Briefly, tissues were kept cold and cleaned to remove traces of blood. Subsequently, tissues were placed on a nylon mesh membrane. After centrifugation, extracellular fluid was collected and cryopreserved until further analyses. Single cell suspensions of tumor samples were prepared using mechanical and enzymatic digestion, as previously described [39]. Transcriptome data from 526 ccRCC and 287 papillary RCC (pRCC) patients were obtained from the Cancer Genome Atlas Database (TCGA, KIRC and KIRP datasets) [40,41] using the UCSC Xena platform [42]. This set provides clinical and survival data over an extended time period. The large cohort size enables a sound statistical analysis of expression data in Cox survival analysis (see below), where each factor has a sufficiently large sample size. Expression is depicted as log2(fpkm-uq+1) values. For single-gene studies, median was used to define high and low values.

### Analyses of bulk and single cell RNA sequencing data

KEGG [43] and MetaCyc [44] databases were used to define genes involved in fatty acid degradation (FAD), fatty acid elongation (FAE), fatty acid synthesis (FAS), and cholesterol synthesis (Chol). Average linkage hierarchical clustering was performed on the Euclidean distances between all FPKM values of gene expression for the respective KEGG sets using the julia package “Clustering.jl”, version v0.14.2 (https://github.com/JuliaStats/Clustering.jl). For better comparability, the clustering was in all cases cut at a fixed number of 20 clusters and proved to be robust against varying linkage and distance types (Fig. S1B). For illustrational purposes, we refer to clusters that represent less than 25 % of patients as “minor”, mostly representing outliers in the large TCGA datasets. Clusters comprising 25 % or more ccRCC cases are called “dominant” and are numbered in decreasing order of overall survival, i.e., cluster 1 has higher overall survival than cluster 2. To assess the robustness of a particular cluster, mean silhouette scores were computed for each dominant cluster, neglecting any minor clusters. The mean silhouette score allows to measure how well data points are grouped into clusters and specifies how each point fits into its assigned cluster compared to other clusters. A higher score (closer to 1) means better clustering [45]. A Cox proportional hazard model was fitted to the survival data available on the Xena platform [42] using the julia package “Survival.jl”, version v0.2.2 (https://github.com/JuliaStats/Survival.jl). One-hot encoding was used to include TNM classification, whereas age in years was modeled linearly. The age and TNM specific patient data are included in Suppl. Table 2. Single cell RNA sequencing data from Bi et al. [46] were analyzed using The Single Cell Portal [47].

### Lipidomics

Total fatty acid analysis was carried out by gas chromatography coupled to quadrupole mass spectrometer (GC-qMS) as described previously [48]. In brief, samples were derivatized to fatty acid methyl ester (FAME) in the presence of internal standards. FAMEs were separated on a highly polar BPX70 column (SGE, UK) using a GC-2010 coupled to a GCMS-QP2010 detector from Shimadzu. Quantification was done in selected ion monitoring (SIM) mode using calibration with authentic standards. For analyses of lipid species, lipid extraction was performed according to the method of Bligh and Dyer [49] in the presence of not naturally occurring lipid species as internal standards. Tissue homogenates representing a wet weight of 2 mg were extracted. Lipid species were measured by direct flow injection analysis (FIA) using a triple quadrupole mass spectrometer (FIA-MS/MS) and a high-resolution hybrid quadrupole-Orbitrap mass spectrometer (FIA-FTMS). FIA-MS/MS was performed in positive ion mode using the analytical setup and strategy described previously [50,51]. A fragment ion of *m/z* 184 was used for lysophosphatidylcholines (LPC) [52]. The following neutral losses were applied: Phosphatidylethanolamine (PE) and lysophosphatidylethanolamine (LPE) 141, phosphatidylserine (PS) 185, phosphatidylglycerol (PG) 189 and phosphatidylinositol (PI) 277. Sphingosine based ceramides (Cer) and hexosylceramides (Hex-Cer) were analyzed using a fragment ion of *m/z* 264 [53]. Glycerophospholipid species annotation was based on the assumption of even numbered carbon chains only. A detailed description of the FIA-FTMS method was published recently [54,55]. Triglycerides (TG), diglycerides (DG) and cholesterol esters (CE) were recorded in positive ion mode as [*M*+NH_4_]^+^at a target resolution of 140,000 (at 200 *m/z*). CE species were corrected for their species-specific response [56]. Phosphatidylcholines (PC) and sphingomyelins (SM) were analyzed in negative ion mode as [*M*+HCOO]^-^ at the same resolution setting. Multiplexed acquisition (MSX) was applied for the determination of free cholesterol (FC) and the respective internal standard (FC[D7]) [56].

### Oil red O staining

Cryopreserved tissues from tumors and adjacent healthy kidney were cut on a cryotome and stored at −80 °C. After drying and PBS washing, tissues were stained with Oil Red O (ScienCell, #0843-SC), according to manufacturer’s instructions. Subsequently, nuclei were stained using the Hemalaun staining kit (Merck, #1.09249.0500), and slides were fixed using the Faramount Aqueous Mounting Medium (Dako, #S3025), according to the manufacturer’s instructions. Representative images were captured using the ECHO Rebel-System microscope.

### Cell culture and flow cytometry

Tumor immune cell infiltration was assessed as described previously [39,57]. For flow cytometric analyses, cells were stained with the following reagents: anti-CD3, anti-CD4, anti-CD8, CD25 (all BD), Perforin (BioLegend), Ki-67 and Fixable Viability Dye eFluor 780 (both eBioscience). To assess nutrient uptake, cells were stained with BODIPY 500/510 C1, C12 (Thermo Fisher). Treatments with oleate and palmitate were performed as follows: single cell suspensions from ccRCC tumor were cultured in RPMI medium (Gibco) supplemented with AB-serum (Corning), Glutamax (Gibco 35,050,061), pyruvate (Gibco 11,360–039), B-ME (Gibco 31,350–010), PenStrep (Gibco 15,140–122), amino acids (Gibco 11,140–035), and CD3/CD28 coated stimulation beads (cell:bead ratio of 1:1; Thermo 11132D) in the presence or absence of albumin (Sigma, A6003)-conjugated palmitate (200 µM, Sigma P0600) or oleate (200 µM, Sigma O1008). After treatment, supernatant was collected and analyzed for IFNγ production by ELISA (R&D DY285B-05). Cells were treated for one day (CD25 and INFγ) or three days (Perforin, Ki-67). Cells were re-stimulated with Cell Stimulation cocktail (eBioscience 00–4975–93), fixed and permeabilized (BD Cytofix/Cytoperm™ 554,714) and stained. Flow cytometry was performed using LSRFortessa (BD). Data were analyzed with the FlowJo software (Tree Star Inc.).

### Statistics

Statistical analysis was performed using GraphPad Prism and the julia packages StatsBase v0.33.14, Clustering v0.14.2, Distances v0.10.7, and Survival v.0.2.2. Differential gene expression was determined after TMM normalization with R 4.0.1 and edgeR [58,59]. Significance was determined using one-way ANOVA with Bonferroni’s post-hoc test (for comparison of 3 or more groups) or using paired or unpaired Student’s *t*-test (for comparison of 2 groups). Comparison of categorical variables was performed using the Chi-square test. Correlations were calculated using the Pearson correlation coefficient. For Kaplan-Meier plots of single genes, the median expression was used to define high and low values and log-rank test was used to compare the survival curves. Significance levels were set as * *p* < 0.05, ** *p* < 0.01, *** *p* < 0.001.

## Results

### Lipid gene expression predicts ccRCC patient survival independently of major clinical parameters

ccRCC tumors engage in lipid-metabolic activity, yet the prognostic relevance of this phenomenon beyond the single gene level remains poorly understood. To address this, we follow a partially supervised approach and select metabolic gene sets from the KEGG and MetaCyc databases [43,44] (Fig S1A). This approach is designed to group patients that have a similar expression pattern of the selected metabolic genes, but may otherwise be completely different, e.g. due to different clinical conditions. The gene sets that allow a grouping in this way (Fig. S1B) can reveal patterns that would otherwise remain hidden in the high dimensional feature space. In some instances, these clusters define patient groups that were predictive for survival, independent of other clinical confounders. The groups are defined by average linkage hierarchical clustering based on the Euclidean distances between the FPKM normalized expression.

**Fig. 1:**
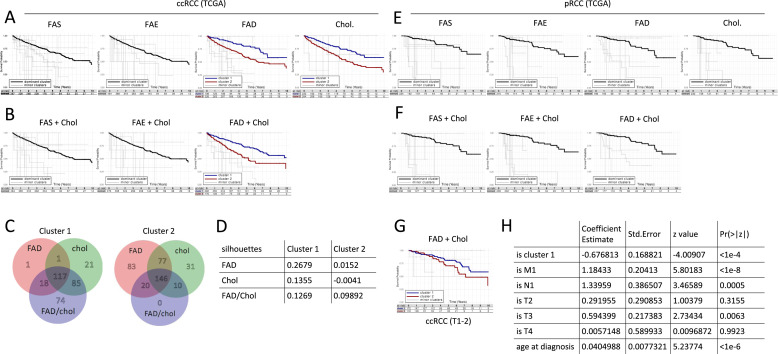
**Lipid gene expression predicts ccRCC patient survival independently of major clinical parameters.** Fatty acid synthesis (FAS), fatty acid elongation (FAE), fatty acid degradation (FAD), and cholesterol biosynthesis (Chol) gene sets were defined using KEGG and MetaCyc databases [43,44]. Transcriptome data was obtained from TCGA datasets clear cell renal cell carcinoma (ccRCC) and papillary renal cell carcinoma (pRCC). Detailed description of the methodology can be found in the Methods section. A cluster dendrogram can be found in Fig. S1B. Clusters comprising 25 % or more cases are called “dominant” and are highlighted (black for one dominant cluster, blue and red for two dominant clusters). Clustering was applied on the ccRCC (**A-D**) and pRCC (**E,F**) patient cohort. (**C**) Venn diagram showing the number of individuals in the prognostically favorable and unfavorable cluster 1 and 2, respectively, depending on the gene sets used for the clustering. Most cluster attributions coincide, albeit 74 patients could be attributed to the favorable cluster 1 only by the combined FAD and Chol gene set. (**D**) Mean silhouette scores of the two dominant clusters were computed to assess the stability of the gene set for clustering. A positive silhouette score indicates that the cluster is consistent and the expression data of all patients within the same cluster is uniform. (**G**) ccRCC tumor data was analyzed as in (B) after exclusion of T3 and T4 tumors. (**H**) A Cox proportional hazard model was fitted to the overall survival data. One-hot encoding was used to include TNM classification of the tumors and if the individual was included in our beneficial cluster 1. Age was modeled linearly in years. The table shows the strata and the contribution of the individual confounders to the overall model, yielding a hazard rate, along with the probability for the null hypothesis that the corresponding confounder does not have an effect on the survival. From the latter, we deduce that the metabolic phenotype detected by our clustering approach ("is cluster 1") is prognostic.

Genes involved in fatty acid synthesis (FAS) or fatty acid elongation (FAE) identified each a single dominant cluster. In contrast, clustering based on genes involved in fatty acid degradation (FAD) and cholesterol biosynthesis (Chol) identified each two dominant ccRCC patient clusters that differed in outcome (Fig. 1A). To test a potential synergism of metabolic pathways, patient clustering was repeated with FAS, FAE and FAD gene sets being combined each with the Chol gene set. Only the combination of FAD/Chol was able to stratify ccRCC patients into two prognostically different clusters (Fig. 1B). While the utilization of both gene sets alone successfully stratified patients to such clusters, 74 patients with a favorable prognosis were identified only when using the combined approach (Fig. 1C). Accordingly, the FAD/Chol combination improved the mean silhouette value of the prognostically unfavorable cluster, showing an improved stability and predictability of the combined approach (Fig 1D). Therefore, FAD/Chol clustering was used for subsequent analyses.

To evaluate the disease specificity of the FAD/Chol clustering model, we assessed its role in papillary RCC (pRCC), the second most common kidney malignancy after ccRCC. A clustering approach based on FAS, FAE, FAD and Chol gene sets failed to identify major prognostically different clusters alone (Fig. 1E) or in combination (Fig. 1F) when studying the transcriptomes of 287 pRCC patients. Metabolism is ultimately linked to cancer cell survival and growth and lipid-metabolic signatures might thus correlate with tumor size and aggressiveness. However, after excluding large ccRCC tumors (T3+T4), the FAD/Chol clustering sustained its predictive power (Fig. 1G). Moreover, multivariate analyses using the Cox proportional hazard model showed that the predictive power of the FAD/Chol clustering was independent of metastatic status, lymph node status, tumor size and age of ccRCC patients (Fig. 1H), indicating that differences in survival are indeed driven by distinct metabolic features revealed by the differential expression of the gene sets under consideration.

Thus, lipid metabolic gene-based outcome prediction was ccRCC specific and independent of major clinical parameters.

### Unfavorable lipid metabolic ccRCC cluster associates with increased *c-Met* copy number and altered tumoral inflammation

**Fig. 2:**
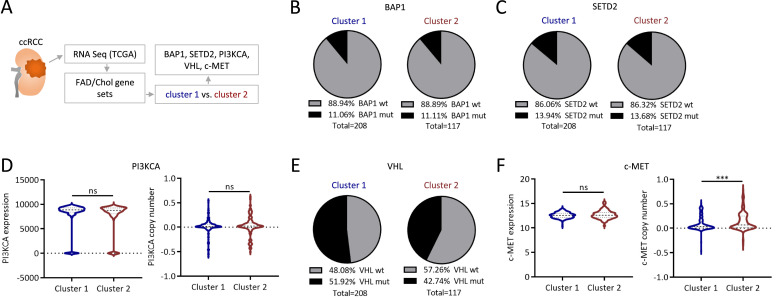
***VHL, SETD2 and BAP1*****mutation status and*****c-Met*****copy number in ccRCC patient clusters defined by lipid metabolic gene transcription.** Expression of fatty acid degradation (FAD) and cholesterol synthesis (Chol) genes was studied using the RNAseq data from the Cancer Genome Atlas (**A**). ccRCC patients were clustered as in Fig. 1 and the prevalence of *BAP1* mutations (**B**), *SETD2* mutations (**C**), the expression and copy number of *PI3KCA* (D), the *VHL* mutation prevalence (*p* = 0.12 in Chi-square test) (**E**), and the expression and copy number of *c-MET* (**F**) were assessed. unpaired Student’s *t*-test, *** *p* < 0.001.

Oncogenes can regulate cellular lipid metabolism [33,60]. Therefore, the relevance of common ccRCC specific oncogenes was assessed in pre-defined metabolic clusters (Fig. 2A). Comparing the two major FAD/Chol clusters, no differences were observed in the prevalence of *BAP1* or *SETD2* mutations (Fig. 2B,C) and in the expression and copy number of *PI3KCA* (Fig. 2D). However, the inferior-survival FAD/Chol cluster (cluster 2) tumors showed a trend toward a lower prevalence of *VHL* mutations (*p* = 0.12; Fig. 2E), while the *c-MET* copy number, but not expression, was increased (Fig. 2F).

Tumor metabolism shapes the tumor micro-environment and directly affects the phenotype and function of tumor infiltrating immune cells [34]. A positive role of intratumoral T cell driven inflammation has been suggested for various tumor entities. Nevertheless, T cell infiltration can also be prognostically unfavorable [[23], [24], [25],^28^,^29^]. We therefore studied the inflammatory landscape of ccRCC and its connection to expression of lipid metabolic genes.

First, standalone inflammatory markers were assessed in ccRCC transcriptome (Fig. S2A) to reveal that none of the studied genes was predictive. Furthermore, high expression of interferon gamma (*IFNG*) was associated with impaired survival of ccRCC patients (Fig. S2A) and infiltration with CD4 and CD8 T cells was higher than in adjacent healthy kidney tissue and further increased in advanced tumors (Fig. S2B,C). The expression of individual genes might not adequately represent the inflammatory context of tumors. Therefore, transcriptome-wide clustering of patients based on pre-defined gene sets of inflammation-related genes (Fig. S2D) was performed next. However, this approach could not distinguish major patient clusters with favorable or unfavorable prognosis (Fig. S2E). A gene set of T cell exhaustion genes (Fig. S2F) identified three prognostically different groups (Fig. S2G,H). An exhaustion signature correlated positively with CD8a expression, but was not different between the lipid metabolic clusters 1 and 2 (Fig. S2I,J).

**Fig. 3:**
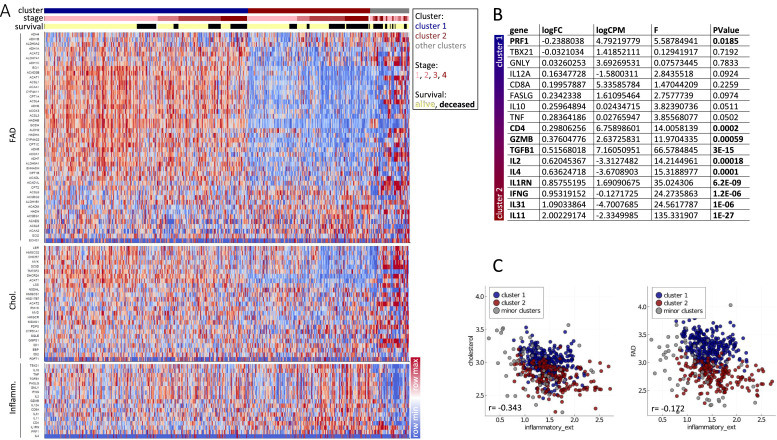
**Metabolic gene clustering reveals an association of tumoral inflammation with lipid metabolism in ccRCC.** (**A**) ccRCC patients were clustered as in Fig. 1. Affiliations with a cluster of lipid genes, disease stage and survival status and the relative expression of selected genes are depicted. The rows were percentile-normalized, where blue (red) indicates lowest (highest) expression within samples. (**B**) Differential expression of inflammatory genes as in (A). Differential gene expression is depicted as logFC, logCPM, F and p values and was determined after TMM normalization with R 4.0.1 and edgeR[58,59]. (C) Gene-set scores, defined as averages across all genes in a set, were calculated for Chol, FAD and the inflammatory gene sets.

Nevertheless, expression of several of the inflammation-associated genes was increased in the inferior-survival FAD/Chol cluster 2, suggesting a broad activation of immune cells in ccRCC tumors with low lipid metabolic activity (Fig. 3A). This trend was opposed by perforin 1 (*PRF1*), a CD8 T cell effector molecule, as its expression was increased in the superior-survival FAD/Chol cluster 1 (Fig. 3B). Studying the relations of gene set scores, defined as averages across all genes in a gene set, we observed a negative correlation of inflammatory genes with metabolic genes, which was more pronounced for the Chol than the FAD gene set (Fig. 3C).

These data demonstrate that clustering of ccRCC patients based on lipid gene expression associates with *c-MET* copy numbers and distinct patterns of tumoral inflammation, while being independent of a T cell exhaustion signature.

### ccRCC tumor lipidomics suggests a dominant role of cholesteryl-oleate

**Fig. 4:**
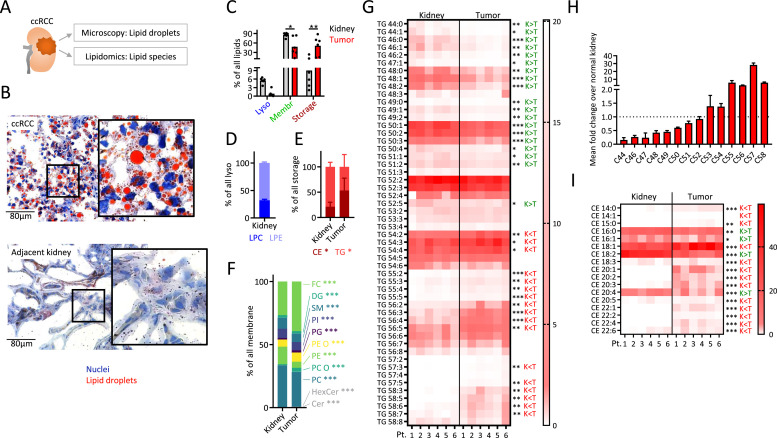
**ccRCC tumor lipidomics suggests a dominant role of cholesteryl-oleate.** (**A**) Schematic. Tumor and normal kidney specimens from ccRCC patients were stained for lipid content using Oil Red O (**B**). Shown are representative images of 14 samples. (**C-F**) ccRCC tumor specimens were analyzed by flow-injection hybrid quadrupole-Orbitrap mass spectrometry to measure the relative amounts of bulk lysosomal, membrane and storage lipid species (C), lysophosphatidylcholine (LPC), lysophosphatidylethanolamine (LPE) (D), cholesteryl ester (CE), triacylglycerol (TG) (E), ceramide (Cer), hexosylceramide (HexCer), phosphatidylcholine (PC), phosphatidylcholine-ether (PC O), phosphatidylethanolamine (PE), phosphatidylethanolamine-ether (PE O), phosphatidylglycerol (PG), phosphatidylinositol (PI), sphingomyelin (SM), diacylglycerol (DG), and free cholesterol (FC) (F). (**G-I**) TG and CE sub-species differentiated according to the number of carbon atoms and carbon-carbon double bonds. Asterisks represent significant differences between kidney and matched tumor samples. Heatmap color represents the percentage of parent species. In (H), the mean fold change of TG of specific carbon lengths in paired tumor/kidney samples is shown. *K* = adjacent kidney, *T* = tumor. Green = group mean of K higher than group mean of T, red = group mean of K lower than the group mean of T. ANOVA with Bonferroni´s post-hoc test (C) and paired Student’s t-test were used to compare normal kidney and matched tumor tissues (D-I).

Transcriptomic data suggested a prognostic role of ccRCC lipid metabolism. To assess, whether transcriptomic signatures also translate to changes in lipids, we performed lipid staining of cryopreserved specimens of tumors and adjacent normal kidney tissues (Fig. 4A). While no lipid accumulation was observed in the latter, ccRCC tumors accumulated large amounts of lipid droplets of various sizes (Fig. 4B). Next, mass spectrometric analyses of matched ccRCC and normal kidney tissues were performed to study the distribution of lipid species. Species typically found in storage lipids were highly increased in tumor specimens, confirming data from Saito et al. [7], while lyso-lipid species tended to be lower in tumor specimens, in agreement with work of Schaeffler et al. [2]. The membrane lipid fraction was lower in ccRCC as compared than normal kidney tissues (Fig. 4C). While lyso-lipids were marginal in ccRCC, a sub-analysis revealed a preference towards lysophosphatidylethanolamines (LPE) over lysophosphatidylcholines (LPC) in normal kidney tissues (Fig. 4D). Storage lipids of tumors consisted of approx. 50 % cholesteryl esters (CE) and triacylglycerols (TG), while CE were significantly lower abundant in storage lipids of normal kidney tissues (Fig. 4E). Comparing lipid classes found in membranes, tumors showed increased fraction of free cholesterol (FC), while phosphatidylethanolamine (PE) and phosphatidylcholine (PC) were decreased (Fig. 4F). Next, we studied the number of carbons and carbon-carbon double bonds (*C* = *C*) in TG species. While TG with shorter carbon chains were characteristic for normal kidney tissue, tumor TG were found to harbor longer carbon chains (Fig. 4G,H). Lastly, tumor CE were predominantly stored as 18:1 (cholesteryl oleate), while kidney CE were preferably 18:2 (cholesteryl linoleate) (Fig. 4I).

Collectively, ccRCC tumors accumulated storage lipids, predominantly as TG and CE, with cholesteryl oleate being dominant.

### Oleate-related species accumulate in ccRCC and negatively correlate with CD8 T cell frequencies

**Fig. 5:**
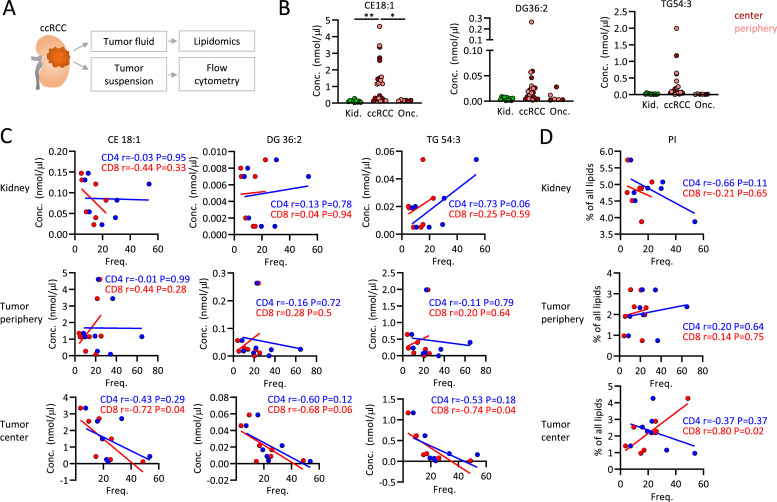
**Oleate-related species accumulate in ccRCC and negatively correlate with CD8 T cell frequencies.** (**A**) Extracellular fluid was extracted from ccRCC tumors and analyzed by lipidomics. Tumor suspensions were assessed for T cell infiltration using flow cytometry. (**B**) Extracellular tissue fluid from ccRCC tumor core and periphery, adjacent kidney tissue, and benign kidney tumors, and lipid species CE 18:1, DG 36:2, and TG 54:3 were measured using mass spectrometry. (**C,D**) Extracellular fluid lipids as in (B) and phosphatidylinositols (PI) were correlated to the frequencies of CD4 and CD8 tumor infiltrating T cells in ccRCC samples. Depicted are Pearson r and P values. Significance levels were set at * *p* < 0.05, ** *p* < 0. 01, *** *p* < 0.001.

Metabolic and transcriptomic studies showed a prognostically relevant connection of tumor lipid metabolism and T cell mediated inflammation. Furthermore, enrichment of some lipid species such as cholesteryl oleate in ccRCC tumors suggested that this connection might be mediated through the tumor microenvironment. In contrast to whole tumor lipidomics (Fig. 4), we studied lipid species in the tumor fluid and their correlations to intratumoral T cells in ccRCC, adjacent kidney and control benign kidney tumors (Fig. 5A).

Consistent with data from whole tumor tissues, we observed an accumulation of CE 18:1, likely cholesteryl oleate, in extracellular space of ccRCC tumors. Furthermore, DG 36:2 and TG 54:3 species, possibly containing oleate (18:1), tended to accumulate in ccRCC (Fig. 5B). Next, the infiltration of ccRCC tumors and normal kidney tissue with CD4 and CD8 T cells was assessed. Interestingly, CD8 T cell infiltration into ccRCC tumor cores correlated negatively with all three oleate-associated lipid species, while no significant correlations were observed for CD4 T cells. No relevant correlations were observed for the tumor periphery, but CD4 T cell frequencies increased with TG 54:3 in kidney tissues (Fig. 5C). These correlations were not observed with oleate-unrelated species such as phosphatidylinositols (Fig. 5D) and phosphatidylglycerols (Fig. S3A).

Thus, extracellular fluid of ccRCC accumulated oleate-related species, which correlated with low infiltration through CD8 T cells. These data support the transcriptomic and metabolic results and point toward lipid metabolism of ccRCC as a regulator of intratumoral CD8 T cells.

### Treatment with oleate decreases function of CD8 T cells in ccRCC

**Fig. 6:**
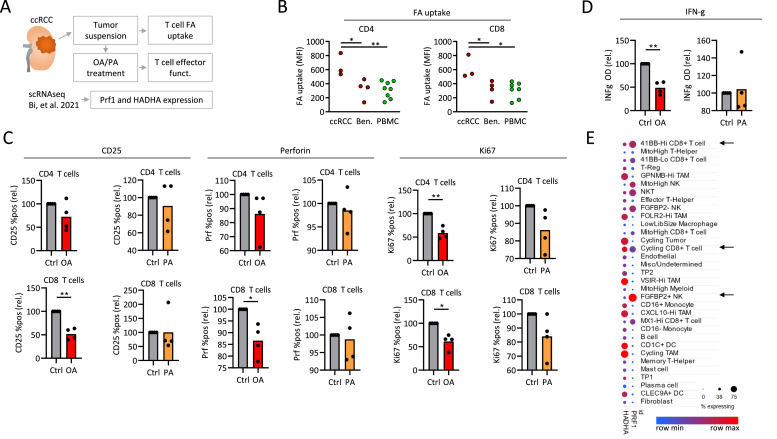
**Treatment with oleate decreases function of CD8 T cells in ccRCC.** (**A**) ccRCC tumors were analyzed for fatty acid uptake capacity of CD4 and CD8 TIL using the BODIPY dye and for T cell activation after treatment with oleate (OA) and palmitate (PA). scRNAseq data by Bi et al. [46]. were analyzed for expression of fatty acid degradation gene HADHA. Fatty acid uptake was measured in CD4 and CD8 T cells from ccRCC tumors, benign kidney tumors (Ben.) and ccRCC patient blood (PBMC) ex vivo (**B**). (**C**) Single cell suspensions of ccRCC tumors were treated with OA or PA and the expression of CD25 (after one day), perforin and Ki-67 (after three days) was measured in CD4 and CD8 TIL using flow cytometry. (**D**) Supernatants of cells stimulated in (C) were assessed for interferon-gamma (IFN-γ) production using ELISA after three days. Values in C,D are normalized to control, paired t-test was used. (**E**) scRNAseq data by Bi et al. [46]. were analyzed for expression of fatty acid degradation gene *HADHA*. Immune cell populations were defined by Bi et al.

The effect of ccRCC lipids on T cells was further addressed by the study of CD4 and CD8 TIL function and single cell RNA expression *ex vivo* (Fig. 6A). CD4 and CD8 RCC TILs from ccRCC showed increased fatty acid uptake as compared to benign kidney tumors (oncocytoma and angiomyolipoma) and peripheral blood T cells (Fig. 6B). Previous analyses suggested a dominant role for oleate-related species in T cell suppression and increased perforin expression was a hallmark of tumor with favorable prognosis (Fig. 3). Therefore, CD4 and CD8 T cells infiltrating ccRCC tumors were cultured in the presence of oleate or palmitate. Oleate, but not palmitate impaired the function of CD8 and to lesser extent CD4 T cells in terms of CD25 and perforin expression and proliferation, measured by expression of Ki-67 (Fig. 6C, Fig. S4A). In addition, production of interferon-gamma was decreased upon oleate treatment (Fig. 6D). Activation and proliferation of peripheral blood T cells was not affected (data not shown).

Studying the single cell transcriptome of ccRCC tumors [46], we focused on the expression of perforin and *HADHA*, a gene involved in oxidation of long-chain fatty acids such as oleate, in different ccRCC infiltrating cell populations. While increased perforin production was observed in CD8 T cells subpopulations with low *HADHA* expression (41BB-Hi CD8 T cells), it was decreased in CD8 T cells with higher *HADHA* expression (Cycling CD8 T cells). The highest perforin-expressing cells were HADHA-low, FGFBP2+ NK cells (Fig. 6E).

Thus, the effects of oleate on ccRCC T cells further supported the transcriptomic and metabolic data by showing that lipid metabolic changes in ccRCC can affect intratumoral T cells. Collectively, lipid metabolism of ccRCC tumors is prognostically relevant and correlates with distinct patterns of T cell mediated inflammation. In vitro studies suggest that lipid species, such as oleate, might negatively affect intratumoral T cells.

## Discussion

RCC tumors are known to be both "immunogenic" and highly metabolically active. Here, we show that clear cell (cc) RCC accumulate lipids and use transcriptomic data to define a ccRCC patient group with prognostically favorable increased tumoral expression of genes involved in fatty acid degradation and cholesterol biosynthesis. This patient group shows decreased intratumoral inflammation and in vitro data suggest a role of tumor fatty acid metabolism in regulation of T cell infiltration and activity.

The prognostic role of lipid metabolic genes in ccRCC has been addressed before. Studies assessing fatty acid synthase [9,10], enoyl-CoA hydratase short-chain 1 (*ECHS1*) [11], HDL-Receptor SR-BI [12], the lipid transporter CD36 [13], FA catabolic enzymes [10,14] or the fatty acid transport protein 4 [15] suggest that these genes regulate ccRCC tumor growth and can predict patient survival. While some studies assessed multiple metabolic genes in ccRCC [6,16], the prognostic role and a possible connection to the metabolic microenvironment of selected lipid metabolic sub-pathways including fatty acid degradation (FAD), fatty acid synthesis (FAS) and cholesteryl biosynthesis (Chol) has not been studied in detail to date. Here, we approached this topic from two opposite directions, studying (1) the underlying genetic and transcriptomic programs, and (2) the resulting products of lipid metabolism in the tissue. As it is unlikely that a single gene regulates lipid metabolism in ccRCC, we selected gene clusters pre-defined by established consortia [43,44]. Even though a direct connection cannot be established, our data suggest that the ccRCC patient cluster with unfavorable prognosis, which displays a decreased expression of several fatty acid degradation genes, accumulates oleate-enriched lipids. In line, this cluster shows decreased perforin expression and perforin was downregulated after in vitro treatment with oleate containing lipids.

Despite our data showing a prognostic relevance of lipid metabolic pathways in ccRCC, the mechanisms that regulate these programs remain insufficiently explored. Yet, our data indicate that oncogenes might contribute. The cluster of patients characterized by increased lipid metabolic activity contained more tumors with *VHL* mutations (by a trend), while the copy number of *c-Met* was decreased. The role of both oncogenes for ccRCC lipid metabolism deserves further evaluation in mechanistic studies. Kumagai et al. have proposed that gastric cancer cells harboring *RHOA* mutations increase FA synthesis and release to support the metabolism of regulatory T cells [61]. These observations from gastric tumors are in line with our data showing that modulation of the intratumoral FA landscape might regulate ccRCC infiltrating T cells.

In contrast to melanoma and several other tumor entities [21,22], T cell infiltration can be negatively prognostic in ccRCC patients [[27], [28], [29], [30]]. Here we observed higher expression of inflammatory genes in the cluster of tumors with inferior survival and down-regulated lipid-metabolic pathways. These genes included cytokines and effector molecules suggestive of a broad immune activation, but interestingly, the sole exception was the expression of *PRF1*. Bearing in mind the overall high T cell infiltration of ccRCC [39,62] and the negative prognostic impact of IFNγ, we propose here that an “unfavorable” T cell inflammation with high levels of IFNγ and low *PRF1* expression might be driven by RCC lipid metabolism. On the other hand, a group of patients with tumors showing high expression of FAD genes and, thus, presumably lower extracellular fatty acids, shows a “favorable” inflammation, hallmarked by *PRF1* expression. This model is supported by our data that discern a subgroup of ccRCC tumors, which accumulate oleate-containing lipids and show low CD8 T cell infiltration, with CD8 T cells being the typical *PRF1* producers. Furthermore, oleate, but not palmitate decreased CD8 T cell function and decreased perforin production in vitro. Data showing low expression of HADHA, a key enzyme involved in oleate metabolism [63] in selected immune populations, while being present in tumor cells and tumor-associated macrophages, further supported the central role of intratumoral oleate. Further studies using single cell sequencing techniques in combination with clinical data will shed additional light on the role of individual metabolic and T cell inflammatory genes in ccRCC. However, cohorts of sufficient size and containing clinical information, such as survival, are currently not available.

If tumoral lipid concentrations affect T cells, the question arises, why the expression of FAS or FAE genes did not perform as strongly as the FAD genes in terms of outcome prediction and correlation with inflammation. We speculate that it is rather the low degradation of lipid species commonly found in serum, such as those with oleate moieties, than a high lipid synthesis that leads to the observed accumulation of lipids in a group of ccRCC tumors. Other than the FAS and FAE gene sets, the Chol gene showed a prognostic relevance. As FAD can lead to increased Acetyl-CoA production, we hypothesize, that the Acetyl-CoA might fuel cholesterol synthesis, rather than the FAS. A possible regulator of this “switch” can be SREBP-2 which induces cholesterol synthesis and, opposed to FAS inducing SREBP-1c, is not suppressed through FA oxidation [64]. However, future studies should address these aspects, as well as a possible connection between systemic and tumor metabolism.

The limitations of our study include a small number of samples in in vitro experiments and metabolomic studies and a short in vitro treatment. Therefore, a general application of our data on the broad RCC patient population is not possible. Furthermore, our study might have neglected the possibility of a small population of T cells with highly tumor-reactive T cell receptors (TCR). TCR clonality of TILs has been repeatedly correlated to patient prognosis but can be highly heterogeneous across different entities [[65], [66], [67]]. For ccRCC tumors, there are few reports that suggest decreased TCR clonality of intratumoral T cells [31], and there is currently no known ccRCC antigen that would allow an identification of tumor specific T cells. In line, Sittig et al. described a surprisingly low number of distinct ccRCC T-cell clonotypes as compared to melanoma [68]. Nevertheless, TCR clonality has not been studied in relation to tumor metabolism and it remains unclear, whether distinct lipid metabolic programs might be used by clonally restricted tumor-promoting and tumor-suppressive T cells.

In conclusion, our study describes a robust clustering approach based on lipid-metabolic gene expression that is both ccRCC-specific and independent of major parameters such as tumor size or aggressiveness. Furthermore, based on tumor lipidomics and in vitro T cell studies, we propose that dysregulation of the lipidome affects intratumoral T cells.

## Ethics approval and consent to participate

All subjects provided written consent according to protocols approved by the ethical committee of the University Hospital Regensburg in accordance with the Declaration of Helsinki.

## CRediT authorship contribution statement

**Jakob Simeth:** Writing – review & editing, Writing – original draft, Methodology, Investigation, Formal analysis, Data curation, Conceptualization. **Simon Engelmann:** Writing – review & editing, Resources, Investigation. **Roman Mayr:** Writing – review & editing, Resources, Investigation, Conceptualization. **Sebastian Kaelble:** Writing – review & editing, Resources, Investigation. **Florian Weber:** Writing – review & editing, Resources, Methodology, Investigation. **Renate Pichler:** Writing – review & editing, Conceptualization. **Katja Dettmer:** Writing – review & editing, Methodology, Investigation, Formal analysis, Data curation. **Peter J Oefner:** Writing – review & editing, Supervision, Conceptualization. **Marcus Höring:** Writing – review & editing, Methodology, Data curation. **Luisa Symeou:** Writing – review & editing, Project administration. **Katharina Freitag:** Investigation, Data curation. **Kilian Wagner:** Investigation, Data curation. **Maximilian Burger:** Writing – review & editing, Resources, Investigation, Conceptualization. **Wolfgang Herr:** Writing – review & editing, Supervision, Funding acquisition. **Marina Kreutz:** Writing – review & editing, Supervision, Funding acquisition. **Rainer Spang:** Supervision, Resources. **Gerhard Liebisch:** Writing – review & editing, Methodology, Data curation. **Peter J Siska:** Writing – review & editing, Writing – original draft, Validation, Supervision, Methodology, Investigation, Formal analysis, Data curation, Conceptualization.

## Declaration of competing interest

Furthermore, all authors declare, that there are no conflicts of interest or competing interests in relation to this submission.

## Appendix Supplementary materials

Image, application 1

application 2 Image, application 2
