# Supplementary material for: Lipid metabolism of clear cell renal cell carcinoma predicts survival and affects intratumoral CD8 T cells
Source: Transl Oncol. 2025 Sep 2;61:102513. doi: 10.1016/j.tranon.2025.102513 (PMC12444183; doi:10.1016/j.tranon.2025.102513)

Fig.S1

A

FAD	
ACAA1	*ADH5*
ACAA2	*ADH6*
ACADL	*ADH7*
ACADM	*ALDH1B1*
ACADS	*ALDH2*
ACADSB	*ALDH3A2*
ACADVL	*ALDH7A1*
ACAT1	*ALDH9A1*
ACAT2	*CPT1A*
ACOX1	*CPT1B*
ACOX3	*CPT1C*
ACSBG1	*CPT2*
ACSBG2	*CYP4A11*
ACSL1	*CYP4A22*
ACSL3	*ECHS1*
ACSL4	*ECI1*
ACSL5	*ECI2*
ACSL6	*EHHADH*
ADH1A	*GCDH*
ADH1B	*HADH*
ADH1C	*HADHA*
ADH4	*HADHB*

FAS
ACACA
ACACB
ACSBG1
ACSBG2
ACSL1
ACSL3
ACSL4
ACSL5
ACSL6
CD36
FASN
MCAT
OLAH
OXSM

FAE
ACAA2
ACOT1
ACOT2
ACOT4
ACOT7
ECHS1
ELOVL1
ELOVL2
ELOVL3
ELOVL4
ELOVL5
ELOVL6
ELOVL7
HADH
HADHA
HADHB
HSD17B12
MECR
PPT1
PPT2
PTPLA
PTPLAD1
PTPLAD2
PTPLB

cholesterol
ACAT1
ACAT2
CYP51A1
DHCR24
DHCR7
EBP
FDFT1
FDPS
GGPS1
HMGCR
HMGCS1
HMGCS2
HSD17B7
IDI1
IDI2
LBR
LSS
MSMO1
MVD
MVK
NSDHL
PMVK
SC5D
SQLE
TM7SF2

B

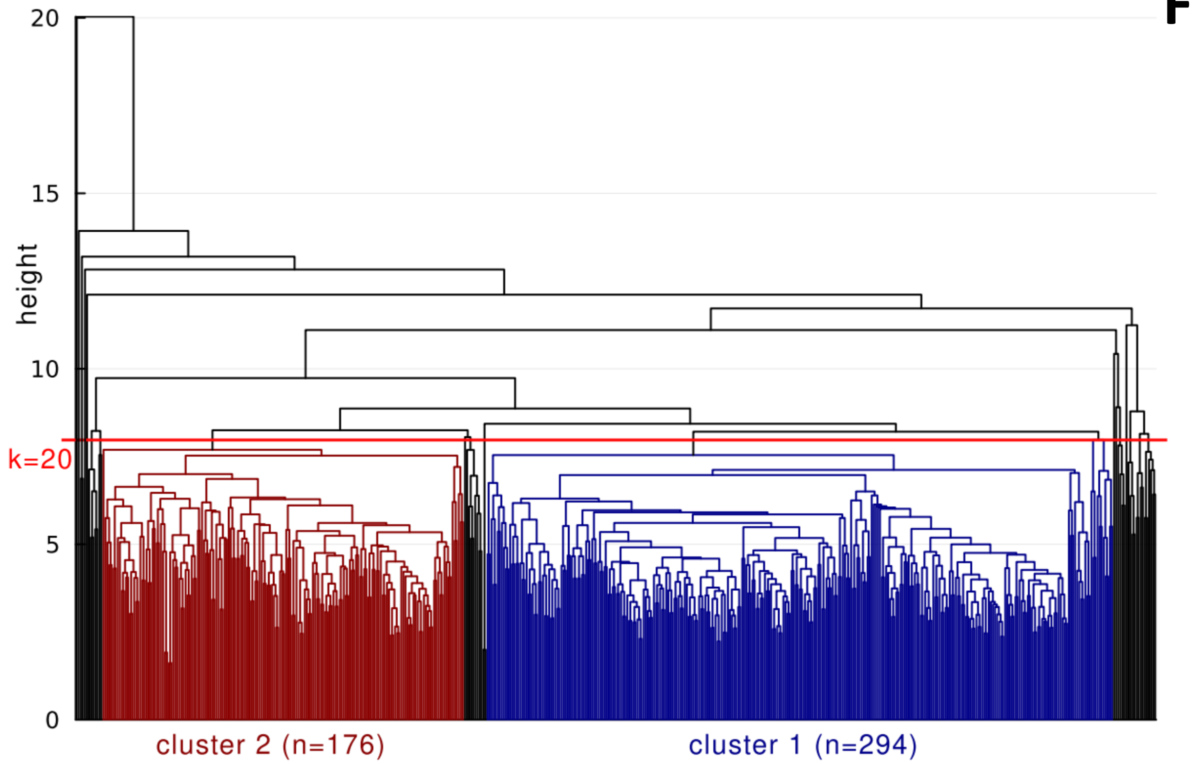

Fig.S2

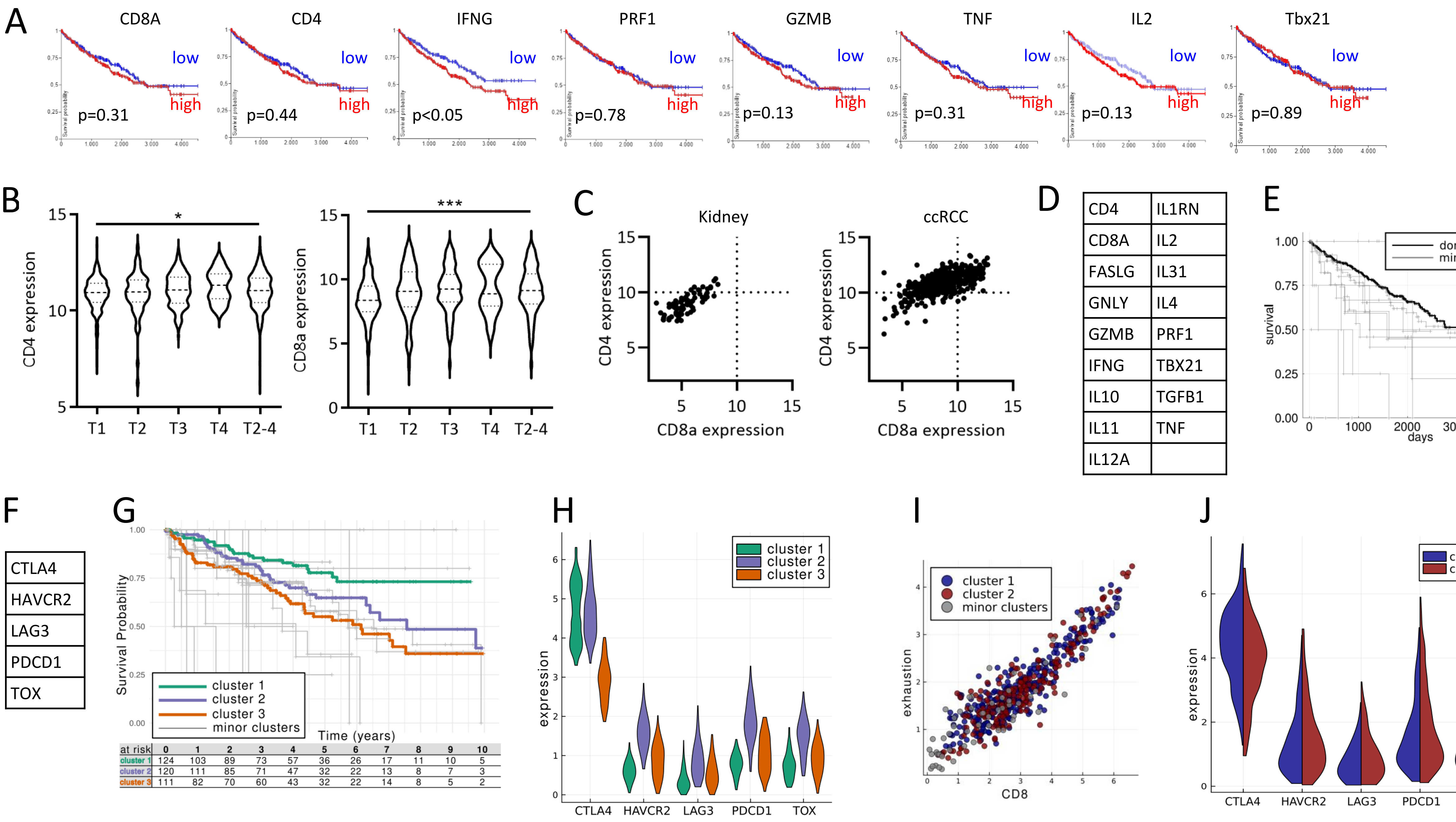
A
CD8A
CD4
IFNG
PRF1
GZMB
TNF
IL2
Tbx21
low
high
p=0.31
p=0.44
p<0.05
p=0.78
p=0.13
p=0.31
p=0.13
p=0.89
B
CD4 expression
CD8a expression
T1 T2 T3 T4 T2-4
C
Kidney
ccRCC
CD4 expression
CD8a expression
D
CD4 IL1RN
CD8A IL2
FASLG IL31
GNLY IL4
GZMB PRF1
IFNG TBX21
IL10 TGFB1
IL11 TNF
IL12A
E
dominant cluster
minor clusters
survival
days
F
CTLA4
HAVCR2
LAG3
PDCD1
TOX
G
Survival Probability
cluster 1
cluster 2
cluster 3
minor clusters
Time (years)
at risk 0 1 2 3 4 5 6 7 8 9 10
cluster 1 124 103 89 73 57 36 26 17 11 10 5
cluster 2 120 111 85 71 47 32 22 13 8 7 3
cluster 3 111 82 70 60 43 32 22 14 8 5 2
H
cluster 1
cluster 2
cluster 3
expression
CTLA4 HAVCR2 LAG3 PDCD1 TOX
I
cluster 1
cluster 2
minor clusters
exhaustion
CD8
J
cluster 1
cluster 2
expression
CTLA4 HAVCR2 LAG3 PDCD1 TOX

Fig.S3

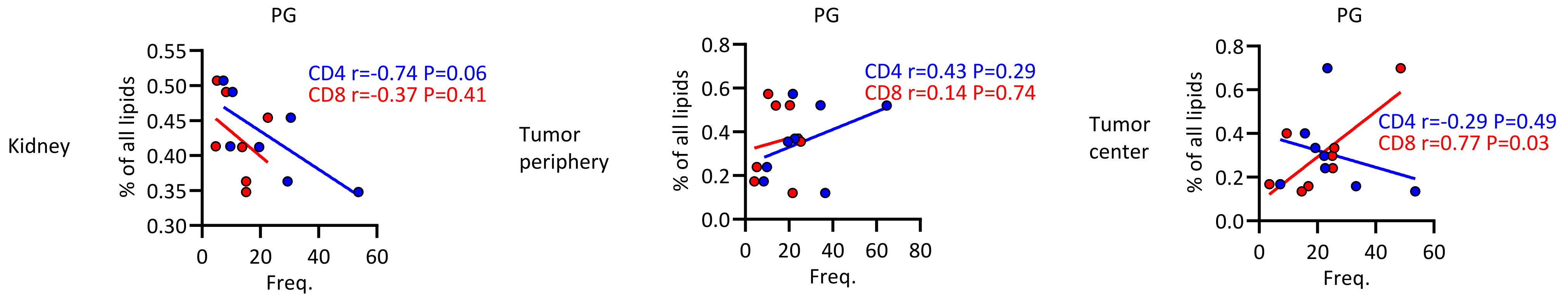

Kidney
PG
% of all lipids
0.55
0.50
0.45
0.40
0.35
0.30
0
20
40
60
Freq.
CD4 r=-0.74 P=0.06
CD8 r=-0.37 P=0.41
Tumor periphery
PG
% of all lipids
0.8
0.6
0.4
0.2
0.0
0
20
40
60
80
Freq.
CD4 r=0.43 P=0.29
CD8 r=0.14 P=0.74
Tumor center
PG
% of all lipids
0.8
0.6
0.4
0.2
0.0
0
20
40
60
Freq.
CD4 r=-0.29 P=0.49
CD8 r=0.77 P=0.03

Fig.S4

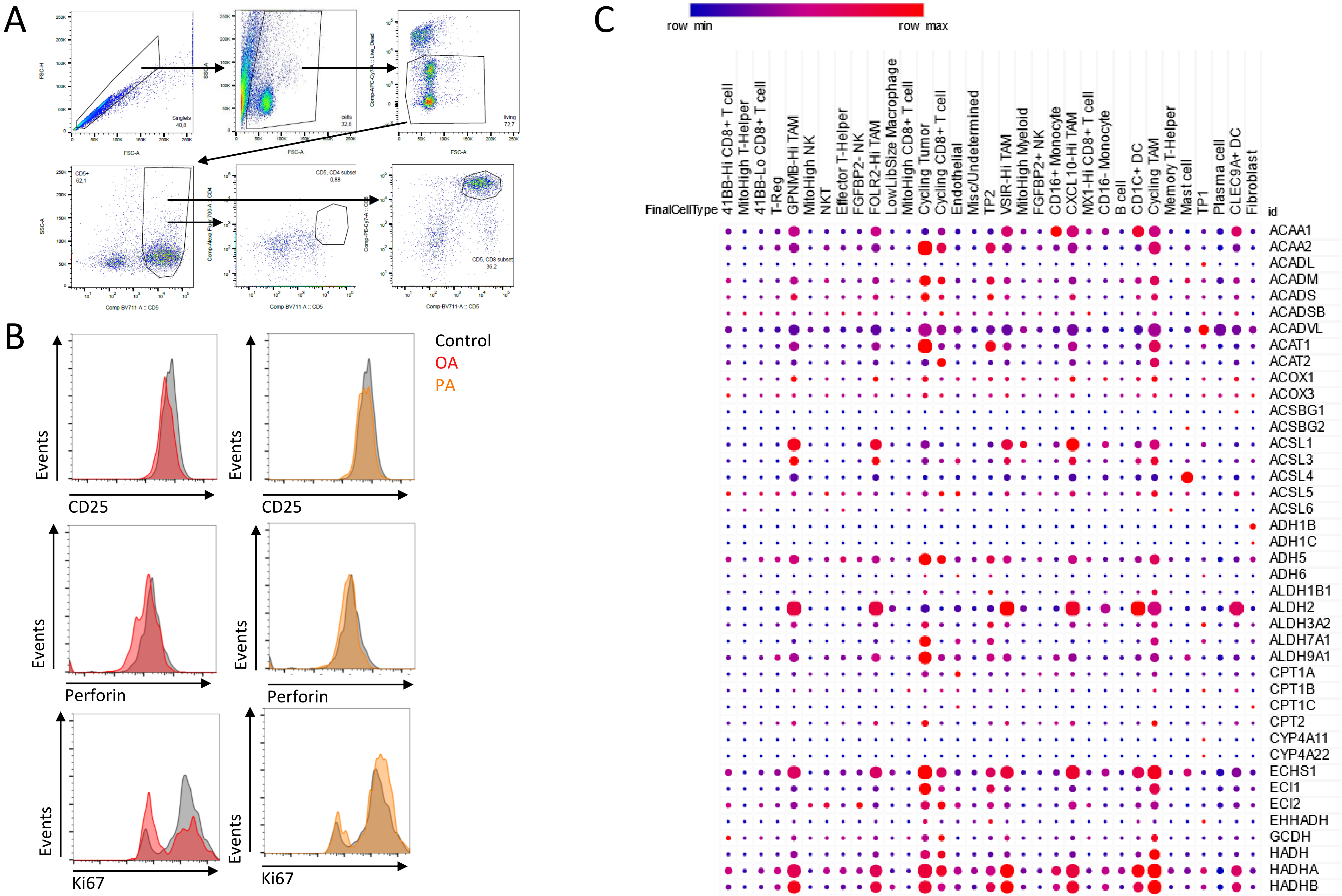
A
Singlets
cells
living
CD5+
CD5, CD4 subset
CD5, CD8 subset
B
Control
OA
PA
Events
CD25
Perforin
Ki67
C
row min
row max
FinalCellType
id

Table S1

Pt.	Gender *	Age (y)	* Histology	T	G
1	m	69	ccRCC	2	2
2	m	57	ccRCC	1a	2
3	m	63	ccRCC	1a	2
4	m	64	ccRCC	1a	2
5	m	71	ccRCC	2a	2
6	f	69	ccRCC	2a	2
7	m	63	ccRCC	1a	2
8	m	91	ccRCC (eo)	1a	2
9	f	82	ccRCC	3a	3
10	m	64	oncocytoma		
11	f	64	ccRCC	2a	3
12	m	57	ccRCC	3a	3
13	f	72	ccRCC	1b	1
14	m	74	oncocytoma		
15	m	55	ccRCC (eo)	3a	2
16	m	66	oncocytoma		
17	m	75	ccRCC	1b	1
18	m	63	ccRCC	1a	2
19	m	69	ccRCC	3a	2
20	f	83	ccRCC	3a	4
21	m	40	ccRCC	1b	1
22	f	77	ccRCC	T1 b	2
23	m	54	ccRCC	T1 a	2
24	m	74	ccRCC	T2	2
25	f	51	ccRCC	T2	3
26	m	52	ccRCC	T3b	2
27	m	76	ccRCC	T3b	2
28	m	73	ccRCC	T3a	2
29	m	52	ccRCC	T3a	2
30	m	65	ccRCC	T3a	3
31	f	77	ccRCC	T3	3
32	f	84	ccRCC	T3a	2
33	f	78	ccRCC	T3a	2
34	m	51	ccRCC	T3a	2
35	f	73	ccRCC	T4	2
36	m	80	ccRCC	T4	3
37	m	60	ccRCC	3c	3
38	m	81	ccRCC	1b	2
39	f	67	ccRCC	2a	1
40	m	78	Onc.	na	na
41	m	49	Onc.	na	na
42	m	60	Onc.	na	na
43	m	70	Ang.m.lip	na	na
44	f	74	ccRCC	1a	2
45	m	72	ccRCC	3a	2
46	f	87	ccRCC	1a	2
47	f	51	ccRCC	3a	2

eo = eosinophil variant

Table S2

Age		
cluster	age *	count
1	2	1
1	3	7
1	4	48
1	5	77
1	6	84
1	7	60
1	8	16
1		1
2	2	1
2	3	6
2	4	28
2	5	47
2	6	52
2	7	36
2	8	6
other	3	2
other	4	11
other	5	14
other	6	15
other	7	12
other	8	2

*** age cohort**

2	20-29
3	30-39
4	40-49
5	50-59
6	60-69
7	70-79
8	80-89

Pathological T		
cluster	T	count
1	1	14
1	1a	87
1	1b	66
1	2	28
1	2a	5
1	2b	3
1	3	3
1	3a	60
1	3b	24
1	3c	1
1	4	3
2	1	5
2	1a	42
2	1b	26
2	2	18
2	2a	4
2	2b	1
2	3	2
2	3a	52
2	3b	22
2	3c	1
2	4	3
other	1	2
other	1a	11
other	1b	16
other	2	8
other	2a	1
other	3a	7
other	3b	6
other	4	5

Pathological N		
cluster	N	count
1	0	131
1	1	4
1	X	159
2	0	79
2	1	10
2	X	87
other	0	29
other	1	2
other	X	25

Pathological M		
cluster	M	count
1	0	239
1	1	36
1	X	18
1	aN	1
2	0	134
2	1	34
2	X	7
2	aN	1
other	0	43
other	1	8
other	X	5

Graphical abstract

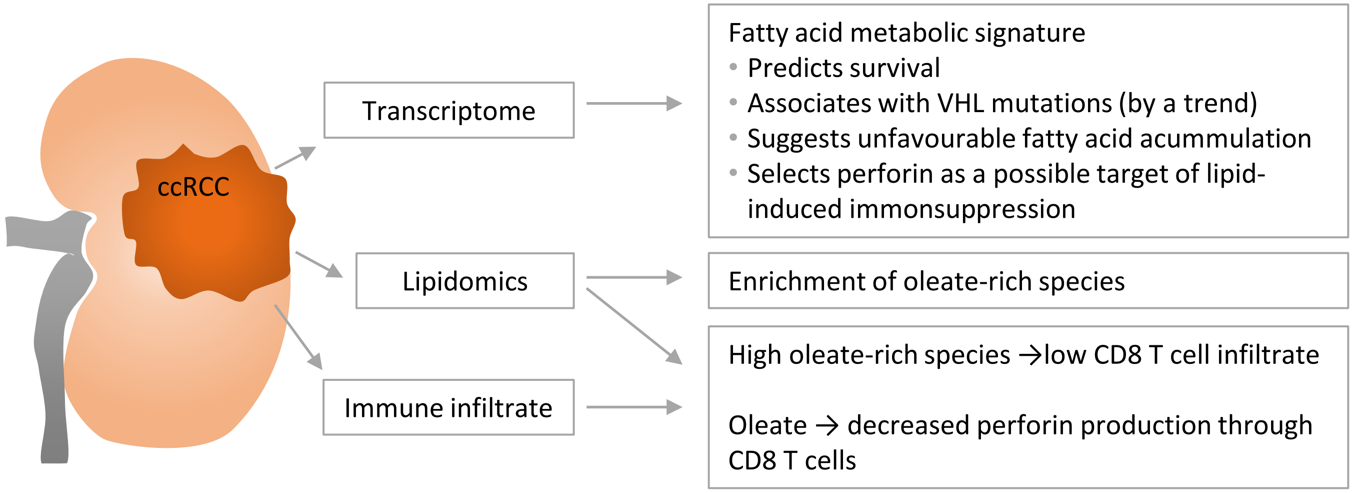

Supplement: Supplementary file 1 [file mmc1.pdf]
