# Supplementary material for: Lipid metabolism of clear cell renal cell carcinoma predicts survival and affects intratumoral CD8 T cells
Source: Transl Oncol. 2025 Sep 2;61:102513. doi: 10.1016/j.tranon.2025.102513 (PMC12444183; doi:10.1016/j.tranon.2025.102513)
Supplement: application 2 [file mmc2.docx]

## Figure S1. Gene sets used for ccRCC patient clustering.

(**A**) Fatty acid synthesis (FAS), fatty acid elongation (FAE), fatty acid degradation (FAD), and cholesterol biosynthesis (Chol) gene sets were defined using KEGG and MetaCyc databases ([43](#_ENREF_43),[44](#_ENREF_44)). (**B**) Dendrogram depicting the patient clustering and the cut-off of 20 clusters.

## Figure S2. Expression of selected inflammatory genes in ccRCC tumors.

(**A**) Expression of selected genes associated with T cell inflammation was assessed in 527 patients using the Cancer Genome Atlas ccRCC cohort. Expression is depicted using log2(fpkm-uq+1) values. For single-gene studies, median was used to define high and low values and the log-rank test was used to compare the survival curves. (**B,C**) CD4 and CD8A expression in T1-T4 ccRCC tumors and in adjacent normal kidney tissue. (**D**,**E**) Clustering of ccRCC patients based on the expression of inflammatory genes and survival. A detailed description of the clustering approach can be found in the methods section. (**F-H**) Patients were clustered as in (E) based on expression of selected T cell exhaustion genes. Expression of individual genes was compared between three clusters. (**I**) Signature of exhaustion genes, as in (F), was correlated to CD8a expression in graphically distinguished metabolic clusters 1 and 2, introduced in Fig. 1. (**J**) Expression of individual exhaustion genes in metabolic cluster 1 and 2 (not significant). Significance levels were set at * p<0.05, ** p<0.01, *** p<0.001.

## Figure S3. T cell infiltration and tumor fluid lipidomics.

Extracellular fluid lipids were isolated from ccRCC tumors (healthy Kidney, Tumor periphery and Tumor center) and concentrations of phosphatidylglycerols (PG) were correlated to the frequencies of CD4 and CD8 tumor infiltrating T cells. Depicted are Pearson r and P values.

**Figure S4. Treatment with fatty acids and scRNA seq data.** (**A**) Exemplary flow cytometry gating of tumor single cell suspensions from ccRCC tissues. (**B**) Exemplary histograms depicting the expression of CD25, perforin and Ki-67 in ccRCC CD8 T cells. (**C**) scRNAseq data and assignment to immune cell populations from Bi et al. were assessed for expression of the fatty acid degradation genes (Fig. 1).
